# Adherence to Oral Antihyperglycemic Agents Among Older Adults With Mental Disorders and Its Effect on Health Care Costs, Quebec, Canada, 2005–2008

**DOI:** 10.5888/pcd12.150412

**Published:** 2015-12-31

**Authors:** Lia Gentil, Helen-Maria Vasiliadis, Michel Préville, Djamal Berbiche

**Affiliations:** Author Affiliations: Helen-Maria Vasiliadis, Michel Préville, Faculty of Medicine and Health Sciences, University of Sherbrooke, and Charles LeMoyne Hospital Research Center, Longueuil, Quebec, Canada; Djamal Berbiche, Charles LeMoyne Hospital Research Center, Longueuil, Quebec, Canada.

## Abstract

**Introduction:**

Nonadherence to oral antihyperglycemic agents (OHAs) leads to an increase in use of health care resources and overall expenditures due to type 2 diabetes and its complications. People with type 2 diabetes are almost twice as likely to have anxiety and depression as the general population. Our aim was to examine health care costs associated with adherence to OHAs and the effect of depression and anxiety disorders on these in older adults with type 2 diabetes.

**Methods:**

We used data from a representative sample (N = 2,811) of community-dwelling adults in Quebec aged 65 years or older who participated in the Étude sur la Santé des Aînés survey. The final sample consisted of 301 participants who were diagnosed with type 2 diabetes and who were taking OHAs. Total health care costs were calculated as the sum of the costs of hospitalizations and outpatient clinic services. Adherence to OHAs was measured using the medication possession ratio. Depression and anxiety disorders were assessed using criteria from the *Diagnostic and Statistical Manual of Mental Disorders, 4th Edition*. We also analyzed data by the Charlson Comorbidity Index, age, sex, education, and marital status, using generalized linear models.

**Results:**

Nonadherence among people without depression or anxiety was associated with higher total health care costs ($4,477; 95% confidence interval [CI], $3,754–$5,201; *P* < .001), as was nonadherence among people with depression or anxiety ($11,124; 95% CI, $9,685–$12,562; *P* < .001).

**Conclusion:**

Improving adherence to OHAs among people with type 2 diabetes, particularly those with underlying mental disorders such as depression or anxiety, can decrease health care costs.

## Introduction

The prevalence of diabetes is increasing worldwide and is a growing public health concern among older adults (1). According to the Public Health Agency of Canada, diabetes affects more than 20% of Canadian adults aged 65 or older (2); medication adherence is crucial for improving clinical outcomes and reducing the social and economic burden (3). The main consequence of poor adherence to oral antihyperglycemic agents (OHAs) is decreased glycemic control, leading to diabetes-related complications, including microvascular and macrovascular diseases (4).

In the United States in 2012, as much as $105 billion in avoidable health care costs was attributed to nonadherence to medications for 6 diseases: hypercholesterolemia, hypertension, type 2 diabetes, osteoporosis, HIV, and congestive heart failure. However, type 2 diabetes and hypercholesterolemia have the highest impact on avoidable health care costs (5). Medication nonadherence is a common problem among older adults with diabetes (6); in a Canadian study, the rate was 86% (7). The literature suggests a direct relationship between medication adherence to hypoglycemic agents and improved glycemic control and reduced health care costs (8,9). Nonadherence leads to an increase in health care resource use and overall expenditure due to diabetes and its complications (3,10).

Furthermore, people with diabetes are almost twice as likely to have anxiety and depression as the general population (11,12). Both depression and anxiety are associated with poor adherence or to medications in chronically ill patients (6). Total health care costs for people with diabetes and depression are estimated to be as much as 4.5 times higher than for people with diabetes alone (13).

Little is known about the effect of mental disorders on medication adherence in relation to health care costs in older adult populations with diabetes. The objective of this study was to examine health care costs from a health care system perspective among people with depression and/or anxiety and the effect of these illnesses on adherence to OHAs in older adults with type 2 diabetes in a publicly managed health care system.

## Methods

Data used in this study originated from the longitudinal Quebec Survey on Seniors’ Health (Étude sur la Santé des Aînés [ESA]). The ESA was conducted from 2005 through 2008 using a probabilistic sample of French-speaking community-dwelling adults aged 65 years or older (N = 2,811); 94% of the Quebec population speaks French. Potential participants living in northern regions of Quebec, Canada, were excluded on feasibility grounds; in 2005, 10% of the older Canadian population resided in these regions. The sampling frame was stratified by 3 geographic areas: metropolitan, urban, and rural. A proportional sample of households was then constituted according to the 16 health administrative regions of Quebec. A random sampling method was also used to select only 1 older adult (aged ≥65 y) in the household. The participation rate in the ESA survey was 76.5%. The project was reviewed and authorized by the ethics committee of the Sherbrooke Geriatric University Institute.

### Procedure

The in-home interviews, which lasted 90 minutes on average, were conducted by health professionals who received a 2-day training. Because memory problems may affect the accuracy of responses to the ESA questionnaire (ESA-Q) and performance on psychological questionnaires, people who obtained a score of less than 22 on the Mini-Mental State Examination were excluded (n = 27) at the beginning of the interview (14–16). Thereafter, people having no moderate or severe cognitive problems were invited to respond to the ESA-Q (n = 2,784). At the end of the interview, respondents were asked to give written consent, allowing the research team to access their health and pharmaceutical services data from the Régie d’Assurance Maladie du Québec (RAMQ),the agency responsible for Quebec’s health insurance plan.

Using the participant’s health insurance number, we linked the ESA survey and individual-level information from the RAMQ’s medical and pharmaceutical service databases and the health ministry’s MED-ECHO (Maintenance et exploitation des données pour l’étude de la clientèle hospitalière) database on hospitalizations. Information on pharmaceutical services included the dispensed drug’s code, quantity, dosage, and length of treatment and the date the drug was dispensed to respondents. The RAMQ physician services database contains data on claims and physician fees paid for consultations and medical services rendered. Data were matched for 2,494 of 2,504 (99.6%) study participants. Participants with private drug insurance plans were excluded (n = 208), because medications delivered to these participants are not registered in the RAMQ pharmaceutical registry under the public drug insurance plan. The study sample consisted of 2,286 patients aged 65 years or older for whom RAMQ and MED-ECHO data were available.

For this study, patients with type 2 diabetes were identified according to criteria used in the National Diabetes Surveillance System (2). People were considered to have type 2 diabetes if they had 2 physician visits on 2 different days within any contiguous 730-day period or 1 hospitalization with a discharge diagnosis of diabetes mellitus code 250 from the *International Classification of Diseases, 9th Revision* (ICD-9) or the *International Classification of Diseases, 9th Revision, Clinical Modification* (ICD-9-CM).

Only patients taking oral hypoglycemic medications were included; the final sample for analysis consisted of 301 patients receiving oral hypoglycemic pharmacotherapy. A preliminary analysis showed that the presence of mental disorders was not associated with the switching of medication or use of hypoglycemic polytherapy (Fisher exact test, *P* > .05).

### Measures

#### Independent variable

Medication adherence was measured using the medication possession ratio (MPR) (17). The MPR was calculated over a 1-year period for patients taking any oral antidiabetic medications or a combination of these medications. The MPR (%) was calculated as the total day’s supply of medication divided by the number of days in the evaluation period, multiplied by 100 ([total day’s supply of medication/no. of days in evaluation period] × 100). The variable medication adherence was dichotomized (MPR ≥80% vs MPR <80%), as has been proposed (18). Six participants had only a 6-month follow-up period and were categorized as nonadherent.

The respondent’s mental health condition was measured at baseline using the diagnostic module of the ESA-Q based on criteria of the *Diagnostic and Statistical Manual of Mental Disorders* (DSM-IV) (19–21). The ESA-Q is similar to the Diagnostic Interview Schedule and Composite International Diagnostic Interview, which demonstrated good reliability and validity (22). In this study, a DSM-IV diagnosis over a 12-month period was made for the following disorders: major depression, minor depression, mania, specific phobia, social phobia, agoraphobia, panic disorder, obsessive-compulsive disorder, and generalized anxiety disorder. Complete definitions of the disorders studied in the ESA are available (23). For the analysis, respondents were classified as 1) having at least 1 probable DSM-IV disorder (depressive or anxiety disorder) or 2) not having a probable DSM-IV disorder (depressive or anxiety disorder) during the 2-year study period.

The respondent’s physical health condition was measured using the Charlson Comorbidity Index (24). This index has a strong monotonic association of an approximate twofold increase in mortality per increment in index level. This index was calculated using medical claims with ICD-9-CM codes for the 12-month period before the interview date and excluded diagnoses related to type 2 diabetes. Other study variables were education (<10 y and ≥10 y), marital status (married or living as a couple, and single/separated/divorced/widowed), age (65–74 y and ≥75 y), and sex.

#### Dependent variable

Our cost analyses took the perspective of Quebec’s public health care system. The calculation of unit costs (in Canadian dollars) was based on summary annual reports for the province of Quebec using a direct allocation method (25). Total health care costs included costs related to hospitalizations, ambulatory visits (outpatient clinic visits and emergency department visits), physician fees, and outpatient medications for a 1-year follow-up. The method used to calculate unit and overall health care costs has been described (26). Briefly, unit costs were based on data in the Quebec Ministry of Health and Social Services annual budget and the AS-471 and AS-478 financial and statistical reports produced by all institutions in the province of Quebec. The cost of hospitalizations was calculated per diem; emergency department visits and outpatient visits in nonprivate offices (ie, public institutions) were valued at cost per visit. Average provincial costs and activity levels for the 2009 and 2010 fiscal years were used.

### Analyses

Data were weighted to ensure that the true proportions of older adults in each geographic area were reflected. The mean and median sampling design effects were 0.94 and 0.95, respectively. Descriptive analyses were conducted to examine the characteristics of participants. Generalized linear regression models with a γ distribution and log link were used to estimate the association between health care costs and type of costs (ambulatory, in-patient, physician fees, medication) and the possible interaction between medication adherence and the presence of common mental disorders (depression or anxiety).

Dummy variables were created in which comparisons were possible between 1) adherent participants without depression and/or anxiety, 2) nonadherent participants without depression and/or anxiety, 3) adherent participants with depression and/or anxiety, 4) nonadherent participants with depression and/or anxiety. To control for confounding, we adjusted the model for age, sex, marital status, education, and severity of physical health conditions (Charlson Comorbidity Index) and OHA exposure in previous year. Statistical significance was set at *P* < .05. Data were analyzed using SAS version 9.1 for Windows (SAS Institute, Inc).

## Results

In this population-based study, 301 (13.2%) participants diagnosed with type 2 diabetes were taking OHAs (Table 1). Most of these participants were adherent (74.4%). Adherent participants were less likely (odds ratio [OR], 0.49; 95% CI, 0.25–0.94) than nonadherent participants to have a probable depression and/or anxiety disorder. Health care costs by adherence and study variables are presented in Table 2.

**Table 1:** Participant Characteristics (N = 301), by Adherence to Oral Antihyperglycemic Agents, Quebec Survey on Seniors’ Health, Canada, 2005–2008

Characteristic	Participants MPR^a^ <80% (n = 77), n (%)	Participants MPR^a^ ≥80% (n = 224), n (%)	Odds Ratio (95% Confidence Interval)	χ^2^ b	*P* Value
**Age, y**					
65–74	39 (50.6)	90 (40.2)	1 [Reference]	2.56	.11
≥75	38 (49.4)	134 (59.8)	1.53 (0.90–2.57)		
**Sex**					
Male	24 (31.2)	82 (36.6)	1 [Reference]	0.74	.39
Female	53 (68.8)	142 (63.4)	0.78 (0.45–1.36)		
**Marital status**					
Married/living as a couple	31 (40.3)	104 (46.4)	1 [Reference]	0.88	.35
Single/divorced/separated/widowed	46 (59.7)	120 (53.6)	0.78 (0.46–1.31)		
**Education, y**					
<10	19 (24.7)	70 (31.2)	1 [Reference]	1.22	.27
≥10	58 (75.3)	154 (68.7)	0.72 (0.4–1.3)		
**Probable depressive or anxiety disorder**					
Yes	18 (23.4)	29 (12.9)	1 [Reference]	4.73	.04
No	59 (76.6)	195 (87.1)	0.49 (0.25–0.94)		
**Charlson Comorbidity Index**					
0	49 (63.6)	155 (69.2)	1 [Reference]	0.80	.36
≥1	28 (36.4)	69 (30.8)	0.78 (0.45–1.34)		

a Medication adherence was measured using the medication possession ratio (MPR). The MPR was calculated over a 1-year period for patients taking any oral antidiabetic medications or a combination of these medications. The MPR (%) was calculated as the total day’s supply of medication divided by the number of days in the evaluation period, multiplied by 100 ([total day’s supply of medication/no. of days in evaluation period] × 100).

b χ^2^ value determined using logistic regression.

**Table 2:** Average Unadjusted Health Care Costs, by Participant Characteristics and Adherence to Oral Antihyperglycemic Agents, Quebec Survey on Seniors’ Health, Canada, 2005–2008^a^

Characteristic	Average Unadjusted Health Care Cost, Canadian $									
	Ambulatory Visits		Inpatient		Medications		Physician Fees		Total Cost (95% CI)	
	MPR <80%	MPR ≥80%	MPR <80%	MPR ≥80%	MPR <80%	MPR ≥80%	MPR <80%	MPR ≥80%	MPR <80%	MPR ≥80%
**Age, y**										
65–74	2,309	1,530	2,579	754	2,843	3,092	847	584	8,580 (6,165–10,955)	5,961 (5,056–6,866)
≥75	3,710	1,077	4,886	620	2,646	2,745	1,192	449	12,433 (8,888–15,978)	4,891 (3,985–5,797)
**Sex**										
Male	3,138	1,432	3,862	714	2,858	2,923	974	548	10,833 (8,198–13,468)	5,617 (4,783–6,452)
Female	2,695	1,203	3,397	677	2,498	3,004	1,114	498	9,705 (6,198–13,213)	5,383 (4,330–6,435)
**Marital status**										
Married/living as a couple	2,757	997	3,135	415	2,363	2,806	987	394	9,243 (6,333–12,153)	4,613 (3,820–5,405)
Single/divorced/separated/widowed	3,164	1,653	4,109	947	3,003	3,080	1,038	647	11,316 (8,391–14,241)	6,327 (5,314–7,340)
**Education, y**										
<10	2,140	1,356	2,490	884	2,401	3,200	1,037	518	8,068 (4,884–11,252)	5,959 (4,701–7,216)
≥10	3,302	1,344	4,148	612	2,867	2,840	1,010	535	11,328 (8,680–13,977)	5,337 (4,578–6,096)
**Probable depressive and/or anxiety disorder**										
Yes	4,058	1,656	6,351	994	3,503	3,006	1,066	520	14,979 (8,937–21,021)	6,256 (4,177–8,335)
No	2,654	1,304	2,854	658	2,498	2,945	1,002	600	9,008 (6,928–11,088)	5,428 (4,746–6,109)
**Charlson Comorbidity Index**										
0	2,368	1,228	2,575	674	2,396	2,585	835	493	8,175 (6,143–10,207)	14,512 (9,744–19,291)
≥1	4,106	1,617	5,715	760	3,359	3,778	1,336	612	4,980 (4,284–5,676)	6,769 (5,351–8,187)

a Medication adherence was measured using the medication possession ratio (MPR). The MPR was calculated over a 1-year period for patients taking any oral antidiabetic medications or a combination of these medications. The MPR (%) was calculated as the total day’s supply of medication divided by the number of days in the evaluation period, multiplied by 100 ([total day’s supply of medication/no. of days in evaluation period] × 100).

Unadjusted estimates (Table 3) showed that nonadherent participants with and without depression and/or anxiety incurred on average higher total health care costs ($14,979 and $9,008, respectively) than did adherent participants with and without depression and/or anxiety ($6,256 and $5,428, respectively).

**Table 3:** Average Unadjusted Health Care Costs, by Adherence to Oral Antihyperglycemic Agents and the Presence of Mental Disorders, Quebec Survey on Seniors’ Health, Canada, 2005–2008

Cost type	Nonadherent With Depression/Anxiety (Reference Group)	Adherent Without Depression/Anxiety	Adherent With Depression/Anxiety	Nonadherent Without Depression/Anxiety
	Mean Cost, Canadian $ (95% CI)			
Ambulatory visits	4,058 (1,374 to 6,743)	1,304 (1,035 to 1,573)	1,656 (754 to 2,559)	2,654 (1,649 to 3,658)
Inpatient	6,351 (−858 to 13,562)	658 (426 to 891)	994 (−64 to 1,923)	2,854 (1,000 to 4,709)
Medications	3,503 (2,392 to 4,613)	2,945 (2,599 to 3,291)	3,006 (2,091 to 3,920)	2,498 (1,863 to 3,133)
Physician fees	1,066 (425 to 1,707)	520 (422 to 617)	600 (303 to 897)	1,002 (657 to 1,346)
Total costs	14,979 (8,937 to 21,021)	5,428 (4,746 to 6,109)	6,256 (4,177 to 8,335)	9,008 (6,928 to 11,088)

After adjustment for study variables (Table 4), among participants without depression/anxiety, nonadherence (vs adherence) was associated with higher total health care costs ($4,447; 95% CI, $3,754–$5,201). Among respondents with depression/anxiety, nonadherence (vs adherence) was also associated with higher health care costs (absolute difference, $11,124; 95% CI, $9,685 - $12,562). Furthermore, nonadherent participants reporting depression/anxiety incurred higher total health care costs ($11,860; 95% CI, $10,697–$13,023) than adherent participants without depression/anxiety. Higher total costs were driven by higher ambulatory and inpatient-related costs, physician fees paid out, and medication costs.

**Table 4:** Adjusted Health Care Cost Differences, by Study Group (Adherence × Mental Health Status),^a^ Quebec Survey on Seniors’ Health, Canada, 2005–2008

Study Group	Nonadherent+ vs Adherent−	Nonadherent+ vs Adherent+	Nonadherent+ vs Nonadherent−	Nonadherent− vs Adherent−	Nonadherent− vs Adherent+	Adherent+ vs Adherent−
	Change in Cost, Canadian $ (95% Confidence Interval)					
Ambulatory visits	3,092 (2,841 to 3,343)	2,687 (2,377 to 2,998)	−1,573 (−1,850 to −1,297)	1,518 (1,362 to 1,675)	1,114 (873 to 1355)	−404 (−615 to −193)
*P* value^b^	<.001	<.001	<.001	<.001	<.001	<.001
In-patient	7,603 (6,771 to 8,436)	7,419 (6,390 to 8,448)	−4,768 (−5,683 to −3,852)	2,836 (2,318 to 3,353)	2,651 (1,854 to 3,448)	−184 (−884 to 515)
*P* value^b^	<.001	<.001	<.001	<.001	<.001	.60
Physician fees	596 (513 to 678)	513 (411 to 615)	−28 (−119 to 63)	568 (516 to 619)	485 (406 to 564)	−83 (−152 to −13)
*P* value^b^	<.001	<.001	.54	<.001	<.001	.02
Medications	569 (438 to 700)	504 (342 to 666)	−1,013 (−1,158 to −869)	−444 (−526 to −363)	−509 (−635 to −384)	−65 (−175 to −45)
*P* value^b^	<.001	<.001	<.001	<.001	<.001	.24
Total cost	11,860 (10,697 to 13,023)	11,124 (9,685 to 12,562)	−7,383 (−8,662 to 6,103)	4,477 (3,754 to 5,201)	3,741 (2,627 to 4,855)	−736 (−1,714 to 242)
*P* value^b^	<.001	<.001	<.001	<.001	<.001	.13

a A plus symbol indicates the presence of depression and/or anxiety; a minus symbol indicates no depression and/or anxiety. The number of patients in each group is as follows: nonadherent with depression and/or anxiety, n = 18; nonadherent without depression and/or anxiety, n = 59; adherent with depression and/or anxiety, n = 29; and adherent without depression and/or anxiety, n = 195.

b *P* values determined using generalized linear modeling.

## Discussion

This is one of the first studies to assess, in a publicly managed health care system, the impact on health care system costs of adherence to OHAs and the presence of depression and/or anxiety in a representative sample of community-dwelling older adults with type 2 diabetes. This study linked health survey data collected during in-home interviews, assessing the presence of probable mental disorders and sociodemographic and economic factors, to health administrative data from the RAMQ on medical and pharmaceutical services use. This linkage increased the validity of results by reducing recall bias on health services use and controlling for various confounding variables.

In this study, 74.4% of participants with type 2 diabetes were adherent to their OHAs; this percentage is similar to a previously reported adherence rate (86%) among older adults taking the same type of drug over a 3-year period in Canada (7).

Our findings showed a differential association between medication adherence and the presence of common mental disorders in terms of total health care costs. These results are consistent with previous findings reporting that increased adherence was associated with lower total annual health care costs among adults (3). Similarly, one systematic review reported that low medication adherence in the population with diabetes was associated with greater health care costs (10). Studies have also reported that the presence of depression in people with diabetes is associated with higher total health care costs (13). In our study, nonadherence among people without depression/anxiety was associated with higher health care costs ($4,477). Among people with depression/anxiety, nonadherence was also associated with a greater effect on total health care costs (absolute difference, $11,124). When comparing adherent people without depression/anxiety to nonadherent people with depression and/or anxiety, we found greater costs among those with depression/anxiety ($11,860).

When examining health care costs, we showed an association between physician fees paid out and ambulatory, inpatient, and medication-related costs and adherence with and without depression and/or anxiety. Previous reports have similarly shown higher ambulatory, inpatient, and total health care costs among a nonadherent population compared with an adherent one (9,10,27). Hepke et al, using private insurance health employer data in a large population of people with diabetes aged 65 years or older, indicated that any potential cost savings associated with adherence to medication were offset by increased pharmaceutical costs (27). That adherent people had lesser costs in our study may be in part because Quebec has a publicly managed health care system in which most of the older adult population is covered under the public drug insurance plan, and this health policy may have a favorable effect on medication adherence to OHAs and, thus, result in decreased overall health care costs.

Our study has limitations. The medication adherence measure was based on delivered medications. Therefore, this measure is subject to the assumption that a prescription filled equals a prescription taken. However, administrative databases are particularly suited for the evaluation of medication for long-term therapy (28). As mentioned, people using insulin therapy were excluded from this study, because the pharmacy records do not contain information on the variability of insulin regimens on a day-to-day basis. Therefore, nonadherence and health care costs may have been underestimated. Furthermore, this study was conducted in Quebec, which has a public health care system, so findings may not be generalizable to those of other health care systems. Nevertheless, the patterns of adherence were similar to those reported in other studies. Finally, the study population was restricted to a representative sample of French-speaking older adults in Quebec and excluded Inuit and Cree populations living in northern regions of Quebec, whose prevalence rates for diabetes are 3 to 5 times those of the general population (29). The results, therefore, may not apply to these groups.

Greater attention should be given to improve medication adherence among patients with diabetes, because nonadherence is associated with higher health care use and costs. More attention should be given to underlying mental disorders such as depression and anxiety. Furthermore, it is important to consider depression and anxiety in the prevention and treatment of diabetes in the elderly population. Poor glycemic control is often a cause of diabetes-related complications, which is an important component of additional direct medical costs of treating people with type 2 diabetes.
